# Report of the Committee on Scarlatina

**Published:** 1856-02

**Authors:** Wm. H. H. Mason


### *Report of the Committee on Scarlatina.*

Mr. President:

In treating upon Scarlatina your committee do not design to notice the general form, course or treatment of the disease; this is laid down sufficiently plain in almost every treatise upon the theory and practice of medicine. Yet there are some things in its pathology and treatment that may well claim our attention.

First: Is it contagious? This question has been decided by almost all authors in the affirmative, though some have entertained doubts upon this point. Nothing in relation to this malady is of more importance than the establishment of this question correctly in the mind of the practitioner. This is so decidedly plain that it needs no argument here to impress it. If we define contagion to be the contact of poisonous particles emanating from a rotten marsh, or floating in an atmosphere poisoned by any unfortunate and unknown combination of sulphur, phosphorus, carbon and azote, united with hydrogen, as well as that emanating from the diseased surface, then there can probably be no question that it is contagious. But if we define contagion to be from a virus by inoculation, contact or miasm from the diseased body, there would be some doubt. Now in deciding whether Scarlatina is or is not contagious, properly, we are not obliged to take as evidence a disease propagated by any unclean or neglected bed, or any ill-ventilated room; for if we do we shall find almost all diseases contagious. But under ordinary circumstances, with proper cleanliness and ventilation, is it contagious? Some diseases are decidedly so under all circumstances, as small pox, measles, lues venera, &c. Compared with these, Scarlatina does not properly admit of being ranked among the contagious diseases.

We may for practical utility resolve the question into this form: Are children of the same family as safe in the same dwelling with the fever, with ordinary cleanliness, as when separated, yet remaining within the district of the epidemic? In point of expediency it certainly wonld be more proper to advise a separation, for if this is an error it is a safe one. We have what seems to be conclusive evidence of its contagiousness, in the successive attacks of members of the same family; and the physician would subject himself to unpleasant censure if by his advice they had remained together. Nevertheless, *de facto*, would not this same family have received the attack any where within the limits of the epidemic? Families have their peculiar idiosyncracies and are obnoxious to the same prevailing diseases. There is some peculiar atmospheric condition which adapts itself to the constitution of members of the same family, although separated, and developes the same disease simultaneously. Notwithstanding the doubts of the contagion of scarlet fever ordinarily, yet in its more malignant type, and in neglected and filthy rooms, there can be no doubt, it is capable of being propagated.

Scarlet fever has been known only three hundred years as a distinct disease; and it seems almost incredible that a disease of so many characteristic marks, so different, too, from any and all others, should so long have escaped the particular notice of the ancient physicians. It probably did exist prior to this period, but was classed among the other eruptive fevers; yet there is reason to suppose that it had its origin at a much later period than many other eruptive diseases. We have certainly no account of it among the Greeks or Romans. Ingrassias, is said by Dr. Copeland, to have given it its first identity; since which time it has suffered no neglect on the part of medical writers.

Passing over the common form of the fever, your committee would call your attention to its nephritic complication. Every one who has had any thing to do with its treatment must have observed the peculiar tendency to subflammation of the kidneys, which may or may not have resulted in anasarca. This occurs, or rather is made manifest, during the desquamatory period. There is no reason to suppose that there is any peculiar pathological connection between the fever and the kidneys, more than with any other organ. The epithelial lining of all the cavities takes on more or less of the inflammation; in fact, it is a cutaneous and endepermial disease; hence the vomiting, diarrhœa, coriza, ottites, &c.; but from the peculiar anatomical construction of the kidneys any epithelial disease of their parts would have a more disastrous consequence. The *tubuli urini-*

*feri* are so minute that the desquamatory epithelial scales form a mechanical obstruction, giving rise to urinary congestion, which, if not removed, produces a second and higher degree of inflammation and degeneration. Desquamation takes place upon other cavities, but for obvious reasons not with the same effect. We often observe about this period inflammation of the synovial membranes, which, doubtless, is connected with the tesselated epithelis of those parts.

There is more or less inflammatory action upon the tubes of the kidneys from the commencement, but it is not immediately noticed. Neither is it called for until desquamation commences; nor is it always then, for in most cases the scales are easily washed away by the urine without any obstruction. These scales can be discovered in abundance in the urine under the microscope. Neither is it complication confined to the worst form of the fever, for it frequently occurs in Scarlatina simplex, and when the patient had not been confined to the bed or even the room. This fact of itself proves that it is more of a mechanical obstruction than otherwise.

Of course dropsy is the result of such complication. The peculiarity of this form of dropsy is, that it will not pit on pressure, or move by gravity to the most depending parts. It will not run from the eyelids, or from one side to the other, or even into the extremities. It is a sthenic dropsy, ponded up in tissue, possessing all its tonic power by a dam across the kidneys. This may be in consequence of its sudden appearance, coming on unlike other dropsies before debility has taken place. Some authors say it bears a strong resemblance to Bright's Disease, and does actually sometimes result in the same melancholy nephritic degeneration. It may in its present effect be similar, yet there is a wide difference in the cause and cure. One is mechanical, and sudden in its appearance, and is easily cured; the other is not mechanical, is slow and insidious in its progress, and is not easily cured. In both, albumen and epethelial scales are passed in the urine; but the scales are not so abundant in Bright's Disease as in the other, and we do not find oil globules in Scarlatina, which is characteristic of Bright's Disease. The similarity in their symptoms, and frequently in their final termination, does not make it that peculiar affection so aptly described by Dr. Bright. The pathology of this form of the disease indicates its treatment. Cooling diuretics, to wash away the obstruction, is the proper treatment. Nit. pot., jalap, and squills. either alone or combined, if administered promptly, are usually sufficient. The more stimulating diuretics should be avoided. It is always best in every case of Scarlatina, however mild, to have this complication in mind, and administer freely the mild diuretics, especially at the first commencement of desquamation.

In the general treatment but little need be said aside from the ordinary antiphlogistic course. But little danger need be anticipated, except in the malignant form, when prompt and active measures are required, or the patient dies. And it is a melancholy fact, and mortifying to science, that it often proves fatal, regardless of remedies. What seems to be most demanded in the malignant form beside tonics, of which bark is the best, are disinfectants to the fauces—*ch. pot.* prepared in *mur. acid*, and given internally in as large doses as the age of the child will admit, acts charmingly as a disinfectant. The throat should be kept as free as possible of the putrid secretions, by gargles, or from time to time small emetic doses of ipecac. There is sometimes more danger from the absorption of these secretions than from the sloughs. It is frequently the case that it forms a crisis apparently favorable, the tongue becoming clean, followed immediately by a fatal train of symptoms by poison. There is no way to prevent this unhappy terminatian but by proper attention to the throat.

In the conclusion of this report your committee would call your attention to belladonna as a prophylactic and remedy. This medicine was first published as a preventive in Scarlatina by Hahnemann, the celebrated author of "Homœopathy." However much this peculiar author may have been led into error by the brilliancy of his own genius, no one will deny that some good things have been written by him. Neither does the fact that it was first treated upon by him, prove any thing for or against that peculiar system of practice of which he stands as head, or afford any reason why it should not be in general use, providing the fact becomes well established. Belladonna was well known to have sometimes produced a rash, resembling that of scarlet fever. This fact pointing toward his fundamental proposition, *Similia similibus curentur*, led him to the supposition that it was a remedy in this disease. But it in fact only proves that a conclusion jumped at through a false theory does sometimes prove correct; and it is by such accidental facts that false theories are sustained generally.

At the present day, authors are divided in their opinions as to the prophylactic property of this remedy. It is very evident, however, that it has not received that trial by physicians generally as it deserves. For if it has no efficacy, in the quantity used, it is harmless, and it is a safe experiment. Although it may possess the therapeutical properties attributed to it by Hahnemann, yet he was very evidently wrong in its application, and it was not by homœopathic doses that he himself made the discovery of these properties. There is no confidence to be placed in it in infinitesimal doses. Although Watson recommends it in small doses, yet they have no relation to homœopathy.

There can be no rational explanation of its *modus operandi* other than by the peculiar eruptive disease which it produces. It would then come under the same pathological law by which the vaccine virus governs small pox. The manner of administering it is to dissolve three or four grains in one ounce of distilled water, of which from six to twenty drops may be given three times a day to the patient. Its tendency is to prevent the disease, or modify it essentially. And if it will do no more than change *Scarlatina maligna* to its simple form, it forces itself upon the consideration of every practitioner.—*From Transactions of N. H. Med. Society.*

Respectfully submitted,

Wm. H. H. Mason, for the Committee.

